# Pain, psychological distress and health-related quality of life at baseline and 3 months after radical prostatectomy

**DOI:** 10.1186/1472-6955-5-8

**Published:** 2006-11-01

**Authors:** Kerstin Wickström Ene, Gunnar Nordberg, Fannie Gaston Johansson, Björn Sjöström

**Affiliations:** 1 The Sahlgrenska Academy at Gothenburg University, Institution of Health and Care Sciences, Gothenburg, Sweden; 2 Departments of Anaesthesiology and Intensive Care, Sahlgrenska University Hospital, Gothenburg, Sweden; 3 Johns Hopkins University School of Nursing, Baltimore, Maryland, USA; 4 University of Skövde, School of Life Sciences, Skövde, Sweden

## Abstract

**Background:**

Inadequate management of postoperative pain is common, and postoperative pain is a risk factor for prolonged pain. In addition to medical and technical factors, psychological factors may also influence the experience of postoperative pain.

**Methods:**

Pain was measured postoperatively at 24, 48, and 72 hr in hospital and after 3 months at home in 140 patients undergoing radical prostatectomy (RP). Patients answered questionnaires about anxiety and depression (HAD scale) and health-related quality of life (SF-36) at baseline and 3 months after surgery.

**Results:**

In the first 3 postoperative days, mild pain was reported by 45 patients (32%), moderate pain by 64 (45%), and severe pain by 31 (22%) on one or more days. High postoperative pain scores were correlated with length of hospital stay and with high pain scores at home. Forty patients (29%) reported moderate (n = 35) or severe (n = 5) pain after discharge from hospital. Patients who experienced anxiety and depression preoperatively had higher postoperative pain scores and remained anxious and depressed 3 months after surgery. The scores for the physical domains in the SF-36 were decreased, while the mental health scores were increased at 3 months. Anxiety and depression were negatively correlated with all domains of the SF-36.

**Conclusion:**

There is a need for nurses to be aware of the psychological status of RP patients and its impact upon patients' experience of postoperative pain and recovery. The ability to identify patients with psychological distress and to target interventions is an important goal for future research.

## Background

Radical prostatectomy (RP) is a procedure that has been performed with increasing frequency in patients with localized prostate cancer. Although the morbidity associated with this procedure is quite low [1], inadequate management of postoperative pain is common [2-5]. Nearly half of the patients who have an operation report moderate to severe pain after surgery [6], despite an increased focus on pain management programs and the development of new standards for pain management [4]. There are a number of risk factors for prolonged pain after surgery, one of the most striking of which is, indeed, the severity of the acute postoperative pain [7,8]. Extensive postoperative pain after RP has been shown to affect the early recovery [9] and discharge from hospital [5]. Although long-lasting pain is not generally encountered after RP, pain problems 3 months after surgery have been reported [10].

Several techniques for postoperative pain management are available for patients undergoing RP: Continuous epidural analgesia (EDA) is a safe and effective method that is frequently used [11,12], although recent studies [13,14] have found also subdural medication e.g. intrathecal analgesia (ITA) with opioids and local anaesthetics to compare favourably with an EDA technique.

In addition to medical and technical factors, psychological factors may also influence the experience of postoperative pain. Studies exploring the relationship between emotional variables and postoperative pain have highlighted the influence of anxiety and depression [15]. According to Katz et al. [16], state anxiety, i.e. anxiety associated with a dangerous situation, is a predictor of both immediate postoperative and persistent pain. Others have also found depression to be a postoperative pain predictor [15,17], an observation that is consistent with our previous findings [5]. Pre-operative anxiety and depression have been found to predict not only postoperative pain but also the postoperative experience of anxiety and depression [15]. These data suggest that patients who enter the hospital in a certain psychological state are at risk of experiencing aggravated postoperative pain as well as prolonged anxiety and depression.

One way to improve our understanding of the consequences of postoperative pain and functioning after hospital discharge is to measure health-related quality of life (HRQOL) [18]. There are many factors that may influence HRQOL after RP. Of these, urinary incontinence and erectile dysfunction represent the major persistent problems after RP that have an obvious negative impact on HRQOL [1]. Although several studies have reported sexual and urogenital problems after RP, relatively little is known about the impact of anxiety and depression on pain and HLQOL after RP. Therefore, the present study was conducted to investigate patients' experience of pain and psychological distress, their HRQL, and the interrelationships between these factors at baseline and 3 months after RP.

## Methods

### Design/Sample

The study was a prospective, longitudinal descriptive study conducted from December 2002 to June 2004. After approval was obtained from the Ethical Committee of Sahlgrenska University Hospital, Sweden, patients undergoing RP were recruited from two hospitals: a University hospital with two surgical units and a community hospital. Three weeks before surgery, patients on the waiting-list for RP, received a letter with written information about the study. Patients willing to participate signed and returned a consent form. The patients answered baseline questionnaires preoperatively and reported their postoperative pain experience. At 3 months after the operation, patients were given a second questionnaire to answer.

### Instruments

#### Demographic form

The demographic form contained questions about age, marital stage, education, employment, and time on the waiting list. Data related to physical status classification according to the American Society of Anaesthesiologists (ASA score), pain treatment, and length of hospital stay (LoS) were collected from the patients' records. Three months after surgery, the patients answered a questionnaire about their worst and mean pain experience, the anatomical location of the pain, and their pain medication (analgesics and for how long administered) at home.

#### Visual Analogue Scale (VAS)

Pain was measured with a visual analogue scale (VAS, 0–100 mm), on which the patients' pain intensity was represented by a point between the extremes of "no pain at all" and "worst pain imaginable." The simplicity, reliability, and validity of this instrument have made the VAS a good tool for describing pain severity or intensity [19].

Pain scores were divided into three broad categories based on pain intensity, as suggested by Bodian et al. [20]. Pain group I was defined as patients whose "worst pain" was scored as VAS ≤ 30 (mild pain) during all 3 postoperative days. Pain groups II and III were defined as patients whose "worst pain" was scored as VAS 31–70 (moderate pain) or >70 (severe pain), respectively, for one or more of 3 subsequent postoperative days. After 3 months, "worst pain" scores were divided into the same categories, based on the "worst pain" level at home.

#### Hospital Anxiety and Depression scale (HAD)

The HAD scale [21] has been found to be a reliable (Cronbach's alpha > 0.80) instrument for assessing the symptom severity of anxiety disorders and depression in somatic, psychiatric and primary care patients and in a general population [22]. The instrument is a 14-item, self-administered rating scale that produces two sub-scales, one measuring anxiety (HAD-A) and the other measuring depression (HAD-D). Each item has four response categories, reflecting a continuum of increasing level of emotional distress. Thus, HAD ≤ 7 indicates no anxiety (HAD-A) or depression (HAD-D), HAD 8 – 10 indicates possible anxiety or depression, and HAD ≥ 11 indicates probable anxiety or depression.

#### SF-36

The SF-36 measures perceived health status by assessing eight health components:

1) *physical functioning *– limitations in physical activity, including self-care activities; 2) *role-physical *– work and activity limitations due to physical problems; 3) *bodily pain *– limitations due to pain; 4) *general health *– overall self-rated health; 5) *vitality *– energy versus fatigue; 6) *social functioning *– limitations in social activities due to emotional problems; 7) *role emotional *– work and activity limitations due to emotional problems; and 8) *mental health *– emotional symptoms (e.g. nervous, depressed). Standardized scores range from 0 (poor functioning) to 100 (good functioning). In addition, a single item addresses the health transition over the past year. The reliability for the Swedish version of the SF-36 is > 0.70 [23].

### Procedure

Three weeks before surgery, consecutive patients on the waiting list for RP received a letter with written information about the study. Patients willing to participate signed and returned a consent form. At the same time the patients answered the form about demographics and the SF-36 questionnaire. The HAD scale was answered the day before surgery. The patients' postoperative pain experience was determined at 24, 48, and 72 hr by asking about "worst pain" during the last 24 hr at rest or when moving. Three months after the operation the patients were mailed the SF-36 and the HAD questionnaires and a form asking about pain at home, together with a stamped return envelope.

Initially, EDA was the routine treatment for postoperative pain in these RP patients; about a year after the beginning of the study, the method for postoperative analgesia was shifted to ITA. Study patients who were deemed unsuitable for either EDA or ITA received systemic opioids for pain relief.

### Statistical analysis

SPSS (version 12.0) for data analysis was used to analyze the data. Continuous variables are presented as means and standard deviation, and categorical data are presented as number and percent. To measure the differences before and after surgery, the paired sample t-test was used. For correlations between variables, we used Pearson's product moment correlation and Spearman's rank order correlation [24]. All tests were conducted at the 5% significance level.

## Results

**Figure 1:**
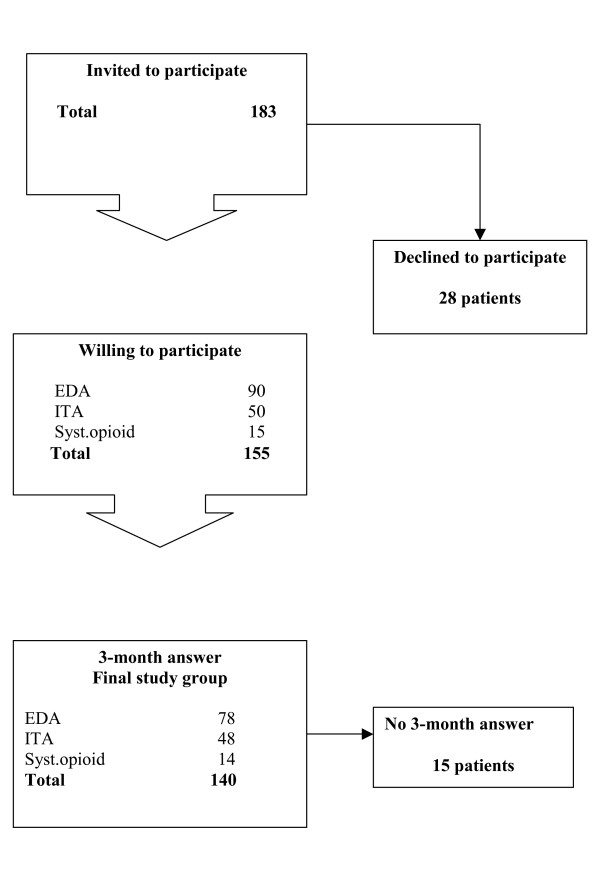
Flowchart for the study, during the period December 2002 to June 2004.

Of the 183 consecutive patients who were invited to participate, 155 patients (85%) gave informed consent to participate. At 3 months after the operation, the patients were mailed the follow-up questionnaires, which were answered and returned by 140 patients (90%) (Fig. 1). The patients with missing questionnaires were equally distributed among the three postoperative pain categories: five with mild, five with moderate, and five with severe postoperative pain.

### Demographics

The mean age of the patients was 63.1 years (range, 43–73). Most of the subjects (91%) were married, and about a third had an elementary education. Half of the patients were retired. After being diagnosed with prostate cancer, 41% of the patients had to wait >3 months for their operation (Table 1).

**Table 1:** Patient demographics and background characteristics (n = 140)

Age	63.1 ± 5.2
ASA class	
I	49 (35)
II	84 (60)
III	7 (5)
Civil status	
single	13 (9)
married/cohabiting	127 (91)
Education	
elementary school	47 (34)
junior high school	28 (20)
senior high school	30 (21)
university	31 (22)
unspecified	4 (3)
Employment	
full-time	58 (41)
part-time	8 (6)
retired	70 (50)
on sick leave	4 (3)
Time on waiting list	
< 1 month	15 (11)
1–2 months	42 (30)
2–3 months	26 (18)
> 3 months	57 (41)

Continuous data are presented as means ± SD and categorical data as n (%).

### Postoperative pain and pain at home

Of the 140 patients who answered the questionnaire at 3 months, 78 (56%) patients had received EDA, 48 (34%) ITA, and 14 (10%) systemic opioids for their postoperative pain relief. There were more patients in the EDA group than in the other two groups who experienced severe pain, both in hospital and at home (28% vs. 14–15%; Table 2). Patients with previous experience of postoperative pain (n = 94) expected high pain scores (p < 0.01), although this level of pain was not actually experienced.

**Table 2:** Differences among pain treatment methods with regard to "worst pain" scores postoperatively and at home (n = 140)

Pain level VAS	Mild ≤30	Moderate 31–70	Severe >70	Total
Postoperative pain	45 (32)	64 (46)	31 (22)	140
EDA	25 (32)	31 (40)	22 (28)	78
ITA	15 (31)	26 (54)	7 (15)	48
Syst. opioids	5 (36)	7 (50)	2 (14)	14
				
Pain at home	100 (71)	35 (25)	5 (4)	140
EDA	53 (68)	20 (26)	5 (6)	78
ITA	36 (75)	12 (25)	0	48
Syst. opioids	11 (79)	3 (21)	0	14

EDA = epidural analgesia, ITA = intrathecal analgesia, Syst. opioids = systemic opioids. Data are presented as n (%).

With regard to the first 3 postoperative days, 45 patients (32%) reported mild, 64 (45%) moderate and 31 (22%) severe pain for one or more days (Table 2). Mean "worst pain" scores, when measured by day, was found to be 40 on day 1, 32 on day 2, and 19 on day 3. There was a correlation between high postoperative pain scores in the hospital and the length of hospital stay (p < 0.01), as well as high pain scores at home (p < 0.01).

Forty patients (29%) reported moderate (n = 35) or severe (n = 5) pain during the 3 months at home (Table 2). When asked about present pain at 3 months after surgery, only three patients reported pain scores above 30. After discharge from the hospital, skin incisional pain (n = 35) and/or diffuse abdominal pain (n = 11) were the most commonly reported. About half of the patients (48%) used analgesics, mostly paracetamol, at home.

### Anxiety and depression

The HAD questionnaire was answered before and at 3 months after surgery by 123 patients (88%). Prior to surgery, 28 men (23%) suffered from possible or probable anxiety (Table 3), but this number decreased to 10 patients (8%) at 3 months after surgery (p < 0.01). There was a correlation between anxiety before surgery and at 3 months (p < 0.01, r = 0.53). Patients with previous experience of postoperative pain scored higher on the preoperative HAD anxiety scale (p < 0.01, r = 0.32). There was a correlation between preoperative anxiety and "worst pain," both in the hospital (p < 0.05, r = 0.23) and at home (p < 0.01, r = 0.26).

**Table 3:** Anxiety and depression in patients (n = 123) before and 3 months after radical prostatectomy

	Before surgery	3 months after	p-value
Anxiety (HAD score)	5.0 ± 3.7	3.0 ± 3.3	<0.001
Depression (HAD score)	3.0 ± 3.3	2.6 ± 3.3	<0.05
HAD-subscale A			
No anxiety	95 (77)	113 (92)	
Possible anxiety	19 (16)	4 (3)	
Probable anxiety	9 (7)	6 (5)	
HAD-subscale B			
No depression	110 (89)	112 (91)	
Possible depression	10 (8)	8 (7)	
Probable depression	3 (2)	3 (2)	

HAD ≤ 7 indicates no anxiety or depression, HAD 8 – 10 indicates possible anxiety or depression, and HAD ≥ 11 indicates probable anxiety or depression. Continuous data are presented as means ± SD and categorical data as n (%).

The number of patients reporting depression decreased from 13 (11%) preoperatively to 11 (9%) at 3 months. Preoperative depression was correlated with "worst pain" in the hospital (p < 0.01, r = 0.23) and "worst pain" at home (p < 0.01, r = 0.31). Patients with the highest pain scores after discharge from the hospital were also the most depressed at 3 months (p < 0.01, r = 0.30), and patients with preoperative depression were the most depressed at 3 months (p < 0.01, r = 0.58).

### HRQOL

At 3 months after surgery, 84 patients (60%) had reached baseline in all HRQOL components except vitality. The physical functioning and role-physical components had significantly decreased (p < 0.001) when compared to baseline, while mental health had increased (p < 0.001) (Table 4). A high pain level during the first 3 postoperative days was correlated with bodily pain (< 0.01, r = 0.26) at 3 months. Preoperative depression negatively affected all components of the SF-36, while preoperative anxiety affected all but physical and social functioning. At 3 months, anxiety and depression were negatively correlated with all components of the SF-36 (p < 0.01).

**Table 4:** Differences in health experiences before and 3 months after radical prostatectomy (n = 140).

**Health areas**	**Before surgery**	**3 months after surgery**	**p-value**
PF	91.3 ± 12.7	85.9 ± 15.6	<0.001
RP	85.5 ± 31.7	65.2 ± 42.3	<0.001
BP	88.8 ± 20.6	89.2 ± 19.8	ns
GH	75.8 ± 19.4	75.3 ± 20.4	ns
VT	75.2 ± 20.8	74.5 ± 22.4	ns
SF	86.8 ± 20.2	85.8 ± 21.3	ns
RE	83.3 ± 32.7	82.3 ± 33.3	ns
MH	76.3 ± 20.3	83.1 ± 17.9	<0.001

Health areas: physical functioning (PF), role physical (RP), bodily pain (BP), general health (GH), vitality (VT), social functioning (SF), role-emotional (RE), and mental health (MH). Standardizes scores range from 0 (poor functioning) to 100 (good functioning). Data are presented as means ± SD.

## Discussion

The study has demonstrated that in RP patients, high levels of pain while in the hospital were associated with an increased length of hospital stay and greater pain after discharge. Furthermore, patients who scored high on the preoperative anxiety and depression scales experienced more severe postoperative pain in the hospital and more anxiety and depression at home at 3 months. Anxiety and depression at 3 months affected all the SF-36 components negatively. Patients with a previous experience of moderate/severe postoperative pain expected the postoperative pain levels to be high, although this expectation was not realized.

A VAS score <40 is frequently considered as an acceptable analgesic level. The mean "worst pain" scores on days 1–3 were found to be equivalent to those previously reported immediately after RP [13,25]. Mean VAS values in the range reported by our patients (19–40) therefore suggest a fairly good postoperative analgesia, yet there were a number of patients that were afforded analgesia that could be considered far from acceptable. Actually, when we allocated the patients into the three pain level groups according to Bodian et al. [20], we found that 57, 42, and 24% of the patients suffered from moderate/severe postoperative pain on days 1, 2, and 3, respectively. The pain outcome was largely unaffected by the pain treatment method used. This spectrum of postoperative pain, with about half of the patients experiencing moderate or severe pain for 1 or 2 days postoperatively and some still reporting pain 3 days after surgery, and one in four patients experiencing insufficient analgesia, is not adequately indicated by mean VAS scores alone.

Our study demonstrates that patients who experienced the highest postoperative pain levels also had the longest hospital stay. Others have reported that pain and nausea affect the quality of the recovery in the immediate postoperative period in patients undergoing RP [9]. When poor analgesia directly affects pain relief, recovery, and length of hospital stay, it obviously also has a strong economic impact. However, this kind of relationship needs to be further clarified in future studies.

Patients with the highest pain scores while in the hospital also experienced the most pain at home after discharge, although at 3 months after surgery, the pain seemed to be well controlled, with only three patients (2%) reporting moderate pain. This finding is consistent with previous reports concerning RP patients [13]. The development of chronic pain after surgery has been considered a consequence of poor peri-operative control of pain [7,8]. However, after RP the risk of severe chronic pain seems to be relatively low, regardless of the analgesic regime used and despite the fact that the pain is not well controlled in all patients following discharge from the hospital.

Psychological well-being is of great significance with regard to the experience of pain after surgery, and psychological preparation of patients undergoing surgery has been shown to shorten hospital stays and reduce the need for postoperative analgesics [6,15]. In the present study, preoperative anxiety and depression were associated with high postoperative pain levels both in the hospital and after discharge. Similar relationships have previously been found between preoperative anxiety and pain at one [16] and three months after surgery [26]. It has been proposed that the levels of preoperative psychological distress may be related to expectations of pain, and this expectation in turn could be influenced by previous experience of a painful surgical procedure [15]. In the present study we consistently found that patients with previous experience of postoperative pain were more anxious preoperatively: There was a relationship between anxiety and depression prior to surgery as well as at 3 months after surgery. This finding suggests that patients entering the hospital feeling anxious and depressed tend to experience more postoperative pain as well as more pain at home. These psychological characteristics do not appear to be related to pre-surgical stress *per se*, since the patients remained anxious and depressed beyond the surgery.

A good nurse-patient relationship allows patients to discuss their anxiety and depression. Preoperative nursing interventions have been found to have a positive effect on preoperative anxiety, postoperative pain, and start-out-of-bed activities [27,28]. As a result of reduced economic resources, an enhanced workload has been imposed upon nurses. Lack of time because of staff shortages and increased workload have been found to be the most common barriers to effective pain management [2]. Obviously, such situations can hinder the development of an extended nurse-patient relationship. Given the relationship between anxiety, postoperative pain, and duration of hospital stay that we have identified here (not least from an economic point of view), more aggressive steps should be taken to prevent patients' postoperative troubles. Hutchison et al [29] have developed a model for treatment of psychological distress in cancer patient, which could be suitable for patients having a RP operation. The authors suggest that all cancer patients should be screened for anxiety/depression and then directed to an appropriate level of psychological care.

Because this study was not designed to evaluate urological problems after RP, the prostate-specific SF-36 was not employed. As compared to baseline, the results for the physical dimensions of the SF-36 had significantly decreased by 3 months after RP in our study. However, the mental health scores were significantly higher postoperatively, in agreement with previous results [30]. Demographic factors such as age and education have been found to correlate with HRQOL [31,32], but no such relationships were found in the present study. However, anxiety and depression at 3 months negatively affected all components of the HRQOL. Similarly, when interviewing patients after RP, Hedestig et al[33] consistently found that these men described the feeling of "being a changed man," longing for the life they had experienced before diagnosis. These patients also reported that their thoughts about the future were associated with growing worry, anxiety, or sadness.

The patients in our study population were treated with one of three different pain management techniques. After we determined [5] that EDA was an insufficient method for pain relief after RP, ITA was adopted as the method of choice for pain treatment. Since the primary aim of the present study was to describe the patients' pain experience and not to compare the three techniques, our patients were not randomized or blinded with regard to treatment regime; indeed, blinding an ITA or EDA regime would have been difficult to accomplish without affecting the performance of the technique.

## Conclusion

In the present study, we found that men with the highest pain scores in the hospital after RP also experienced the most pain during the 3 months immediately after discharge. Patients who experience anxiety and depression preoperatively seemed to have higher postoperative pain scores and remained anxious and depressed 3 months after surgery. Physical functioning had decreased, and mental health had increased at 3 months when compared to baseline. Anxiety and depression at 3 months correlated negatively with all components of HRQOL.

The results of this study indicate that there is a need for further education about the psychological consequences of RP and its impact upon patients' experiences of postoperative pain and recovery. Thus, the ability of health professionals to identify patients with psychological distress and to target interventions are highly desirable goals. There is also a need for interventions to be developed to prepare this group of patients for the physical and mental complications most likely to be experienced after surgery.

## Competing interests

The author(s) declare that they have no competing interests.

## Authors' contributions

KWE and FGJ were responsible for the study design. KWE performed the data collection. All authors contributed to data interpretation, helped revise the manuscript, and approved the final version.

## Pre-publication history

The pre-publication history for this paper can be accessed here:


